# Leaflet Stresses During Full Device Simulation of Crimping to 6 mm in Transcatheter Aortic Valve Implantation, TAVI

**DOI:** 10.1007/s13239-022-00614-6

**Published:** 2022-03-01

**Authors:** N. W. Bressloff

**Affiliations:** Faculty of Engineering & Physical Sciences, University of Southampton, Boldrewood Innovation Campus, Southampton, SO16 7QF UK

**Keywords:** Transcatheter aortic valve implantation (TAVI), TAVI leaflet stress, TAVI leaflet durability, Finite element analysis (FEA), Full valve model crimping simulation

## Abstract

**Background:**

With continuing growth in transcatheter aortic valve implantation for the treatment of a failing aortic valve, there is increasing interest in prosthetic valve durability and the potential damage caused to leaflets by stress. Whilst most available research into the computational prediction of leaflet stresses using finite element analysis, FEA, has focussed on variations during dynamic loading, very little appears to have been reported for the impact of crimping, even though awareness of this effect is widespread. Potentially, this has been due to the difficulty of performing full model simulations of crimping to clinically meaningful diameters.

**Method:**

A full model comprising a self-expanding frame, skirt and leaflets has been developed and crimped to a final diameter of 6 mm. A detailed description is provided of the FEA setup, emphasising the importance of the skirt definition needed to successfully crimp to this small diameter. Then, an analysis of leaflet folding and stresses is presented, particularly with respect to the differences produced between leaflet thicknesses of 0.20, 0.25 and 0.30 mm and for bioprosthetic and polymeric leaflet material models.

**Results:**

In all cases, peak stresses occurred close to the modelled suture lines joining the leaflets and the skirt and high stresses were also present along axially aligned folds in the leaflets. Stresses were lower for the polymeric leaflets.

**Conclusion:**

Successful simulation of crimping requires a finely resolved skirt mesh. Leaflet stresses during crimping are dependent on leaflet thickness, material properties and the ratio of leaflet volume to the available volume inside the crimped valve.

**Supplementary Information:**

The online version contains supplementary material available at 10.1007/s13239-022-00614-6.

## Introduction

Degenerative heart valve disease is a large and growing global health concern. In England alone, for people over the age of 65, the prevalence of heart valve disease is expected to increase from 1.5 million people in 2019, to double that in 2046.^25^ Of the four valves in the heart, the high pressure aortic valve is particularly vulnerable to attritional degeneration. The most common form of degenerative aortic valve disease is known as aortic stenosis (AS). 50% of patients with severe AS die within two years of being diagnosed, if left untreated.^26^

The conventional form of treatment for AS is surgical aortic valve replacement, (SAVR), involving open heart surgery. In recent years, based upon positive comparative data from a series of high quality randomised trials,^22^ transcatheter aortic valve implantation (TAVI) has grown in popularity. For example, in 2019 in the United States, the number of TAVI procedures exceeded SAVR for the first time—72,991 vs. 57,626.^11^ Now, approximately 200,000 TAVI procedures occur per year across the European Union and North America combined and this number is projected to rapidly increase to approximately 300,000 procedures in the short term, as regulatory approvals continue to expand TAVI eligibility for low risk and younger patients.

## TAVI Device Simulations

The growth in TAVI numbers has been reflected in the increasing interest in computational modelling of the TAVI procedure. The majority of reported simulations of both balloon expandable and self-expanding TAVI valves have not used full valve models comprising frame, skirt and leaflets, particularly when simulating the complete procedure of crimping and deployment. Further, as usefully reported by Luraghi *et al*., including a summary of TAVI simulations up to 2019,^20^ of those studies that had simulated full devices, very few had included clinically meaningful crimping diameters. Luraghi *et al*. crimped the CoreValve Evolut R valve to a diameter of 9 mm, Kandail *et al*. only crimped to 15.5 mm due to numerical stability issues^15^ and Bianchi *et al*. crimped a SAPIEN valve to 8 mm.^8^ In two other publications^33,35^ there was a lack of clarity regarding the crimping procedure. Xuan *et al*.^35^ appear to have only crimped a 29 mm valve to a smaller diameter of 26 mm whilst Wu *et al*.^33^ employed an unspecified partial crimping step.

There is some justification for not including both skirt and leaflets in simulations of TAVI, especially when the focus is on the behaviour and performance of a deployed valve. Indeed, exclusion of valve leaflets was justified by Bailey *et al*. who demonstrated that the deployed diameter of a SAPIEN XT valve varied by 0.236% of the frame diameter when comparing the inclusion and exclusion of leaflets.^6^ Despite the usefulness of the simplification and the associated simulation time saving, it is worth noting that Bailey *et al*. were only able to crimp to a diameter of 10 mm. That said, in his PhD thesis, Bailey described crimping simulations of a complete SAPIEN XT model to a small diameter of 6 mm but found it necessary to remove the contact definitions between the skirt and other components including the leaflets.^5^ Similarly, Gunning *et al*. completely excluded the skirt from the crimping step of a self-expanding valve, justifying this on the grounds that the skirt had a significantly lower stiffness than the frames.^13^ These limitations of the respective models hint at the importance of the skirt definition for successful simulation of crimping of a full device.

Rocatello *et al*. justified using a frame and skirt model without leaflets^29^ by citing Mao *et al*.^21^ who had reported a similar finding to Bailey *et al*.^6^ in that the inclusion of TAVI leaflets had a negligible effect on the interaction between the valve and the aortic root. In common with several other published articles,^3,13,27^ Mao *et al*. mapped a skirt and leaflet model into a deployed valve, in preparation for further analysis.^21^ In contrast, Bosmans *et al*., simulated multiple self-expanding frame deployments without skirt or leaflets and a skirt was shrink-wrapped onto the frame post deployment in an investigation of valve leakage.^9^ When crimping just a valve frame, it is relatively straightforward to achieve clinically meaningful diameters as shown by Nappi *et al*. in which models of CoreValve and SAPEIN were crimped to 6 mm and 4.7 mm, respectively.^24^ Bianchi *et al*. simulated crimping of a CoreValve model to 14 Fr—without skirt or leaflets—but then mapped the skirt and leaflets to the deployed frame such that the whole valve model was employed in flow analysis.^8^

Interestingly, one of the earliest published simulations of the valve-in-valve procedure used a model of the SAPIEN balloon expandable valve frame to explore the feasibility of the procedure, highlighting the risk of obstruction of the left ventricular outflow tract and of the coronary arteries. The TAVI model did not include a skirt or leaflets.^10^

## Crimping Procedure

Whether or not full TAVI models have been included, nearly all reported studies have simulated crimping by using radial displacement boundary conditions applied to a cylindrical surface that displaces a concentrically aligned valve using appropriately configured contact definitions between the surface and the valve. Radial displacement can be achieved in a similar way using an array of planes aligned around the circumference of the valve.^32^ Whilst both approaches closely represent the action of a radial displacement crimping machine, more commonly used for balloon expandable devices, they are significantly different to the way in which self-expanding valves such as the CoreValve family of valves are crimped using funnels and relative axial displacement. This more realistic representation has been used more recently but, again, not through a full crimping range.^17,20^

## Leaflets and Leaflet Stresses

Whilst acknowledging the validity of excluding leaflets from TAVI simulations for a range of studies, a key driver for the current study concerned the need to include TAVI leaflets in simulations of crimping so as to assess leaflet stresses and the implications for device durability.^30,31^ Alavi *et al*. have confirmed experimentally that crimping inflicts significant damage to pericardial leaflet tissue that persists over time.^2^ Therefore, if potentially damaging elevated stresses are to be minimised, better understanding of leaflet design, interactions and behaviour is needed during crimping as well as from the loading cycle of the beating heart once deployed. Potentially, leaflet damage that occurs during crimping could produce micro-tears. Subsequently, these will be vulnerable to fatigue caused by alternating stresses generated in the leaflets during cyclic dynamic loading.

Also, there are less obvious potentially damaging effects such as leaflet flutter and how it relates to leaflet thickness as reported by Johnson *et al*.^14^ Several computational studies have investigated the distribution and variation of stresses in deployed leaflets. Xuan *et al*. summarised several of them.^35^ Typically, predicted peak stresses have varied between 1.0 and 3.0 MPa across a range of scenarios. Further, the impact of leaflet thickness on mechanical stresses has been investigated. Thinner leaflets have been consistently shown to lead to higher leaflet stresses, significantly higher during diastole,^1^ with peak stresses close to commissural attachments.^34^

The majority of computational simulations mentioned here have used shell elements for the leaflets and a range of thicknesses have been used, ranging from 0.24 to 0.50 mm. In a study of the effect of a distorted SAPIEN XT frame on leaflet stresses, Bailey *et al*. used leaflet hexahedral brick elements with three elements through the leaflet thickness of 0.30 mm.^7^ More detailed leaflet stress analysis was presented in the Bailey PhD thesis.^5^ Large strain brick elements were used by Martin and Sun in an analysis of the potential effect of TAVI under-expansion on valve durability.^23^

Regardless of the methods used to model valve leaflets, no studies have been found that assess leaflet stresses in a full valve model during simulated crimping to small diameters.

Thus, the main aim of this study was to determine the computational setup required to model crimping of a complete, self-expanding TAVI valve to a small diameter of 6 mm. Using this setup, the goal was to assess the effect on leaflet stresses of different leaflet thicknesses for two material property models, one representative of bovine pericardium and the other based on a polymeric material.

## Materials and Methods

To satisfy the overarching aim of this research, this section provides a detailed description of the computational model and the finite element analysis established for the successful simulation of crimping to a diameter of 6 mm using a radial displacement boundary condition on all elements of a cylindrical surface, initially with a radius 0.15 mm larger than that of the frame. All parts of the model were constructed in Rhinoceros 3D version 7 (Robert McNeel & Associates) and these were imported to Abaqus/Explicit R2019 (Dassault Systèmes) with which all simulations were performed. Interestingly, whilst Rhino has been employed extensively in our group, the study reported here represented the first time that the generative algorithm editor for Rhino, Grasshopper, was used as opposed to scripting using Python or Visual Basic. Although further details are not included here, it is noteworthy that, relative to scripting, the generative design approach was found to provide an efficient interface for constructing, developing and manipulating all parts of the valve assembly.

**Figure 1:**
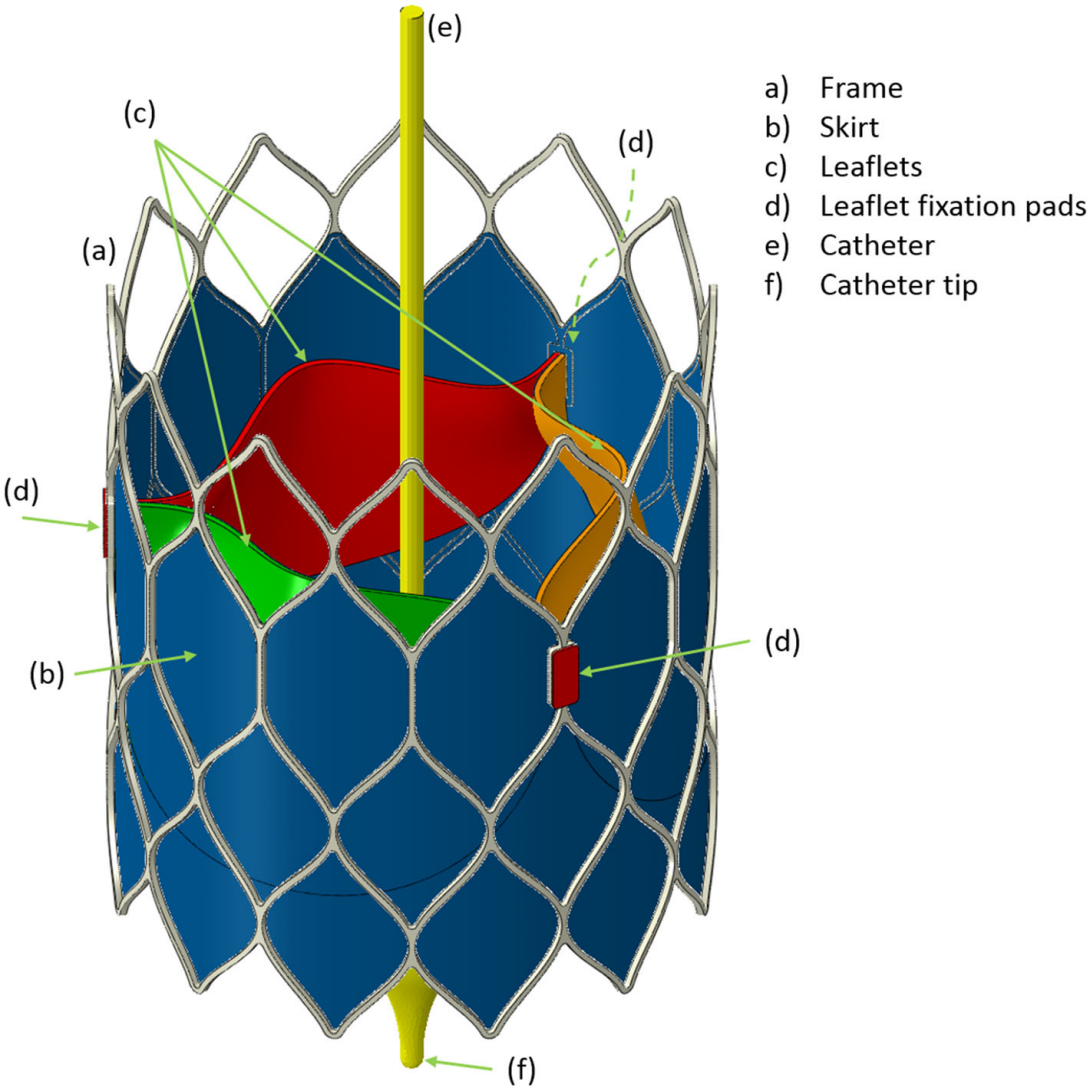
The full, self-expanding valve model assembly showing the frame, skirt, leaflets, leaflet tabs and the catheter.

Figure 1 depicts the self-expanding valve assembly used in this study. Each part of the assembly is now described in turn.

### Frame

The valve frame comprised cell shapes similar to those for Medtronic’s CoreValve but the leaflets were secured to the frame through slots positioned on vertical struts. The leaflet fixation pads were incorporated as a means for securing the leaflets to the frame without using a direct constraint between the leaflets and the frame. The top of the leaflet slots were at a height of 20 mm and the highest row of cells had the same dimensions as the lowest row, leading to a total frame height of 31 mm. The outer diameter of the frame was 26 mm. The author recognises that this frame design might appear to neglect key requirements for frame height, particularly in relation to coronary access, but it has been specified in this way since it is based on a design concept being developed that has sensitive intellectual property features that cannot be revealed at this time. Either way, it does not impact the central focus in relation to crimping and the leaflets. The frame radial thickness was 0.3 mm and the width of the struts was 0.25 mm. The material was setup for Nitinol based on the shape-memory study of superelastic behaviour by Auricchio and Taylor,^4^ with parameters specified by Finotello *et al*.^12^

### Leaflets, Including Tabs and Fixation Pads

The generative design editor was particularly useful in reverse engineering the shape of the leaflets using the leaflet shape generated by Bailey *et al*.^6^ However, a different method was used here to secure the leaflets to the frame. Instead of using a clip, shortened tabs were generated and constrained to thin layers of leaflet material—the leaflet fixation pads—constrained to the outer surface of the frame surrounding the leaflet slots. The pads had a thickness of 0.15 mm.

The bioprosthetic leaflet material properties were defined as described by Bailey *et al*. using the hyperelastic Ogden model.^6^ For the polymer leaflets, a nonwoven polycarbonate based silicone elastomer was used based on the third-order Ogden model developed by Pfensig *et al*.^28^ For both materials, the density was 1.0 g/cm^3^.

### Skirt

The shape of the skirt was defined by the inner outline of the lower three cells of the frame, with cut-outs for the leaflet tabs. Several experiments confirmed existing experience in favour of shell elements for the skirt. Although Bailey *et al*. used membrane elements,^6^ successful crimping to 6 mm, using membrane elements, could not be achieved in the research reported here. Treating the skirt as a solid was also attempted but, whilst it was visually appealing to view the skirt thickness, experiments found that solid elements experienced excessive distortions leading to simulation failure. Based on the experiments undertaken in this research, the definition of the skirt properties, thickness and finite element mesh were deemed to be critical for simulating crimping to small diameters. A wide range of material properties have been used to define the skirt or cuff in previous studies. In particular, Young’s modulus and thickness values have varied between 1 MPa and 0.4 mm^20^ to 500 MPa and 0.01 mm.^6^ More recently, Pasta *et al*. used a value of 55 MPa for the stiffness but did not state the thickness.^27^ In the current study, the thickness was set to 0.06 mm based on the information in a patent by Edwards Lifesciences for the polyethylene terephthalate (PET) fabric used in the SAPIEN 3 valve.^18^ Then, a series of experiments were performed in which the stiffness of the skirt, in a frame and skirt model, was reduced in steps of 50 MPa from 200 MPa. When the skirt stiffness was 100 MPa and above, the distal frame struts became distorted in the final stages of crimping. Thus, the Young’s modulus in the current study was set to 50 MPa, close to the value used by Pasta *et al*.,^27^ and the skirt was assumed to be a linear elastic material. The density was 1.38 g/cm^3^ and Poisson’s ratio was 0.3.

### Crimping Cylinder

The crimping cylinder was aligned concentrically with the valve at a radius 0.15 mm larger than the leaflet fixation pads.

### Catheter

A 1.5 mm diameter catheter was included with a distal tip. The catheter was held in a fixed position throughout the simulation.

### Solution Setup

The Explicit version of Abaqus has long been recognised as the solver of choice for the complex set of interactions that occur when simulating both stent and heart valve deployment. Typically, a simulation is sub-divided into separate steps in which (i) the contacts between individual parts of an assembly are defined by interactions and constraints and (ii) the dynamics are specified by boundary conditions and applied loads. In the simulations described here, there are no applied loads.

### Steps

The crimping procedure was simulated in a single step with time period of 0.1 s. Kinetic energy was maintained significantly below 1% of internal energy throughout the simulation. Mass scaling was used with a minimum time increment of 1.0e−07 s. Along with a smooth step profile, acceleration was kept below the level needed to avoid excessive element strains and wave speeds.

### Constraints

Surface to surface tie constraints were used for the following: (1) the inner face of the frame and the outer face of the skirt; (2) the bottom edges of the leaflets with the inner face of the skirt, visible as the black lines on the skirt in Fig. 1, exactly where the bottom edges of the leaflets join the inner face of the cylindrical skirt, (3) the side faces of the leaflet tabs with the inner face of the leaflet fixation pads and (4) the outer face of the frame with the leaflet fixation pads.

### Interactions

All contact definitions comprised “hard” contact with the Penalty friction formulation and a friction coefficient of 0.1.

### Boundary Conditions

The dynamics of the crimping procedure was defined by displacement boundary condition on the crimping surface as it was displaced radially inwards by 10.3 mm, reducing the crimped frame diameter to 6.0 mm. Three nodes on the frame at mid-height and uniformly arranged around the circumference were held with zero axial displacement.

### Computational Resources

The Iridis5 supercomputer at the University of Southampton was used for all simulations. The dual 2.0 GHz Intel Skylake processor nodes supported 40-way parallel partitioning of the whole simulation domain. All simulations were run with double precision.

Once the full model was defined, final simulations were performed for both leaflet property models with leaflet thicknesses of 0.30, 0.25 and 0.20 mm. A thickness of 0.20 mm is at the lower end of the range of appropriate bioprosthetic leaflet thicknesses, particularly as evidence suggests that leaflets as thin as 0.20 mm are likely to be adversely affected by flutter^14^ as well as experiencing higher stresses, both phenomena likely leading to accelerated degeneration. The respective models are labelled herein for bovine as B300, B250, B200 and polymer as P300, P250, P200.

## Results

### Meshing

Based on established evidence across several research groups, it was initially attempted to run simulations with a base mesh spacing equal to or close to 0.10 mm in the frame, skirt and leaflets, whilst also guaranteeing that at least three elements were present through the thickness of the frame and the leaflets. Hex-dominated meshing was used for the solid parts and 4-node shell elements for the skirt. The frame was meshed as a complete part without exploiting its rotational symmetry.

Multiple simulation experiments ultimately discovered the need for the following skirt mesh settings: (i) small membrane strains—S4RS elements; (ii) second order accuracy and (iii) a finer mesh spacing than the 0.10 mm spacing originally targeted. For the final simulations, the skirt mesh spacing was reduced to 0.05 mm. Mass scaling with a minimum time increment of 1e−07 s was necessary through the simulations. Mesh verification has not been undertaken since settings are at least as good as state of the art simulations reported elsewhere and significantly finer in the case of the skirt.

Table 1 summarises the element definitions for the frame, skirt and leaflets. The frame mesh spacing was set to 0.09 mm to satisfy the requirement for at least three elements throughout the strut cross-sections. For the leaflets, the number of element layers through the thickness was fixed at a value of three and an advancing front swept mesh was used. Further, curvature control was applied to the frame, skirt and leaflet meshes with maximum deviation factors of 0.04, 0.05 and 0.10, respectively.

**Table 1:** Element details for the frame, skirt and leaflets.

Part	Type	Spacing (mm)	Number	Notes
Frame	C3D8R	0.09	100,932	All parts had second order accuracy and enhanced hour-glass control Small membrane strains were used for the skirt
Skirt	S4RS	0.05	854,651	
Leaflet	C3D8R	0.1	98,526	

A total of 1.30 million elements were used in the final simulations leading to approximately 35,000 elements per domain in the 40 partition simulation. This required between 21 and 22 h to run for the complete set of simulations and approximately 15.5 GB memory was used.

### Folding Patterns and Leaflet Stress

**Figure 2:**
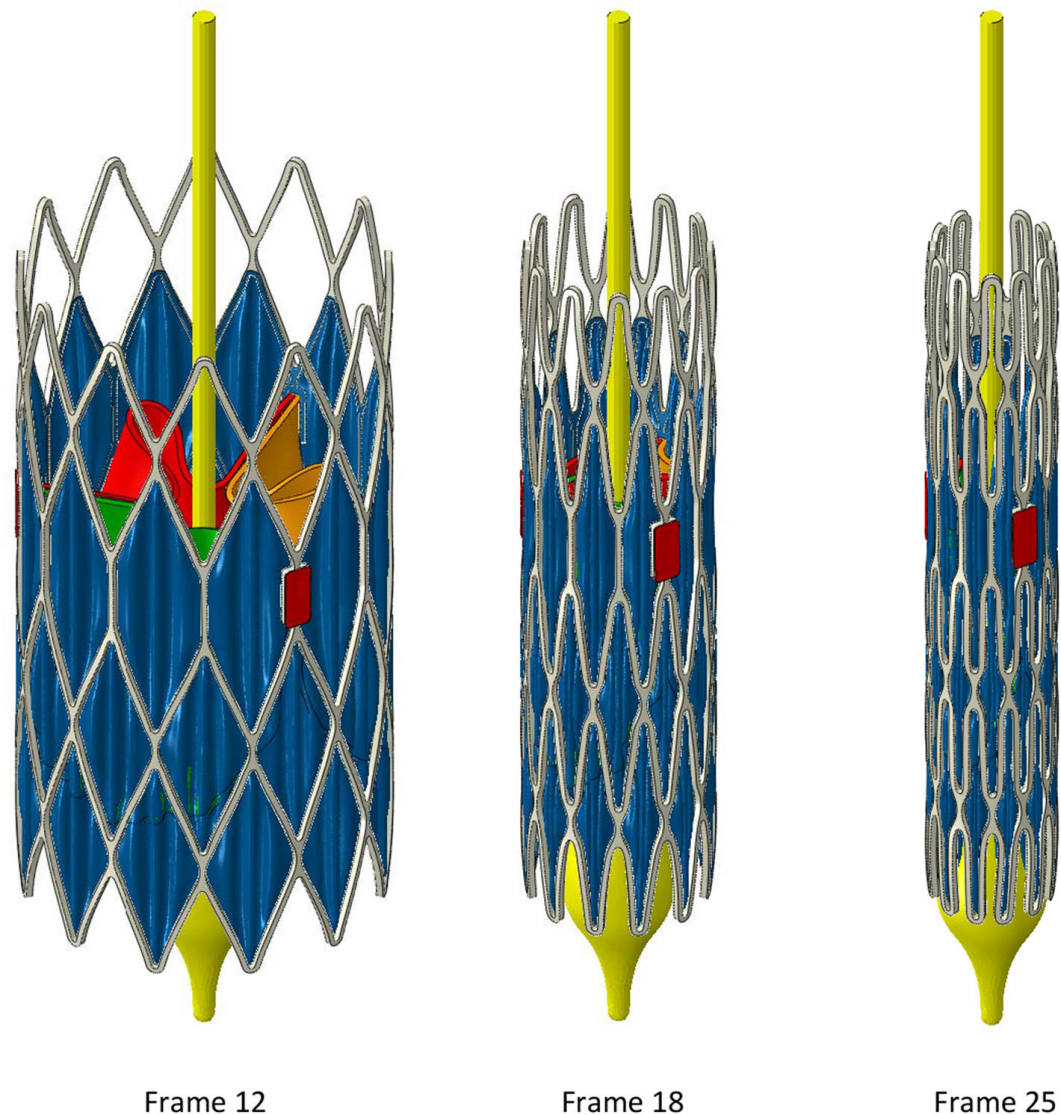
Crimping stages to a diameter of 6 mm.

Isometric views of the B300 valve are shown in Fig. 2 for step frames 12 and 18 and the final step frame 25. The crimping surface has been hidden. The main observations to make concern (i) the regular arrangement of three folds in the skirt within each cell of the frame and (ii) the potential to crimp the frame to an even smaller diameter based on the gaps between adjacent struts in frame 25. However, as shown below, caution is necessary with respect to the impact of tighter packing on leaflet stress, for a given leaflet thickness.

**Figure 3:**
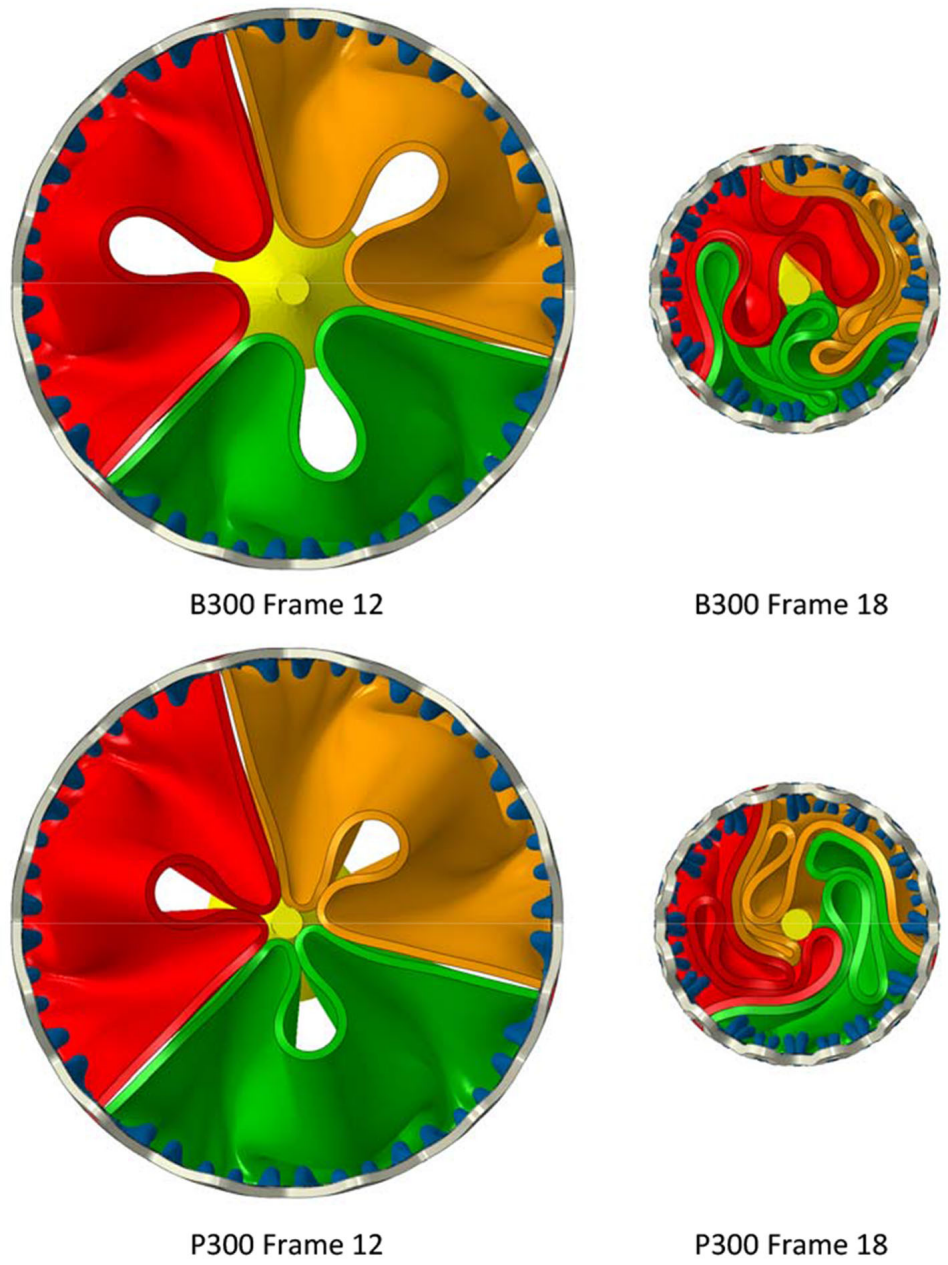
Plan views of B300 and P300 illustrating how the different leaflet models fold during early and intermediate stages of the crimping procedure.

Figure 3 depicts plan views of both the B300 and P300 valves at frames 12 and 18. For both valves, the leaflets exhibit rotational symmetry in frame 12 but this is then largely lost for B300 in frame 18 but maintained for P300. Similar features and differences occurred for the smaller valves and leaflet materials.

**Figure 4:**
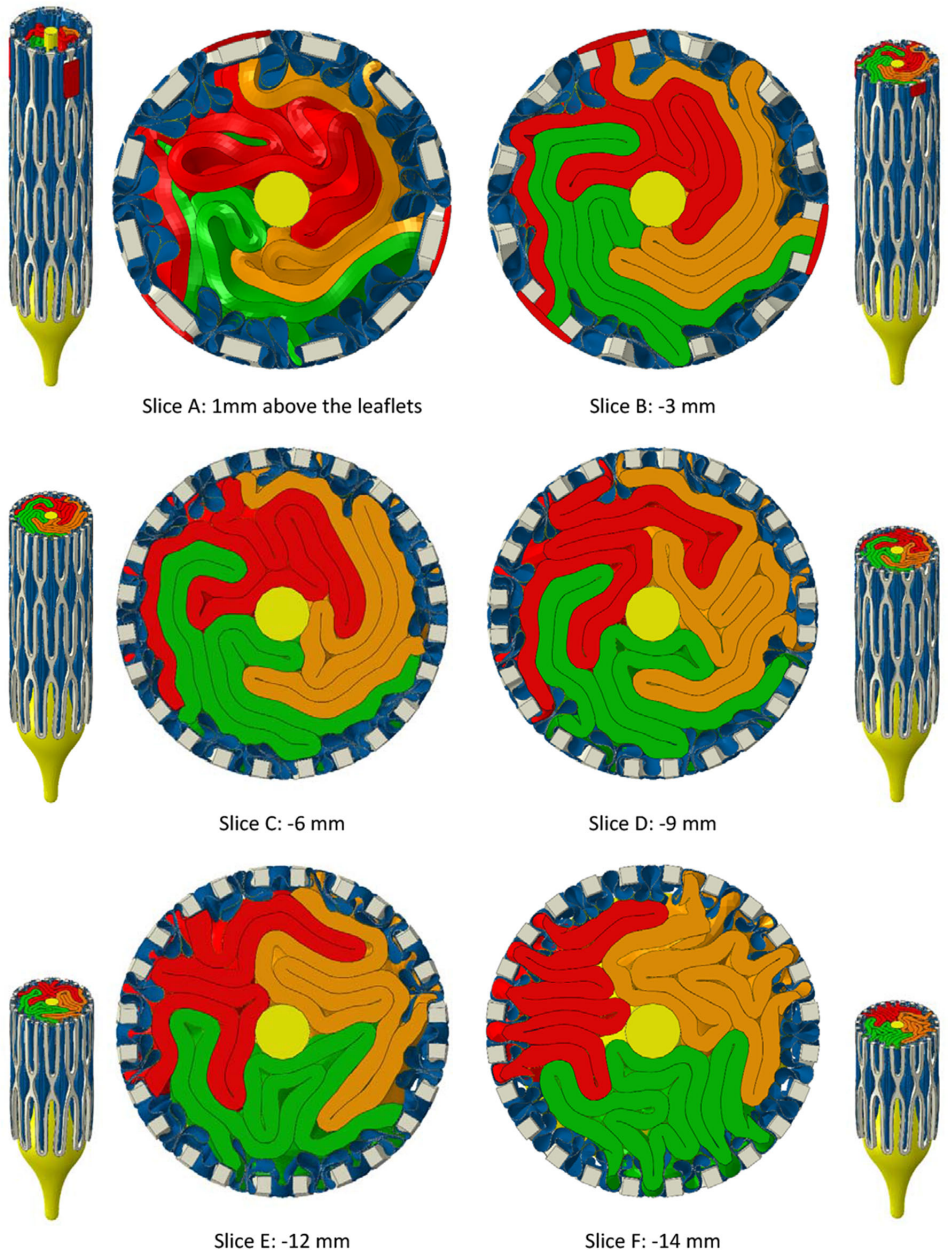
Planar slices through the bovine leaflet model, B300, with 0.30 mm leaflet thickness. The first slice is positioned 1 mm above the top edges of the leaflets. The slices below are spaced at intervals of 3 mm until the last slice—slice F—that is 2 mm below slice E. In this position, the pinching of the leaflets between adjacent frame struts is clear.

**Figure 5:**
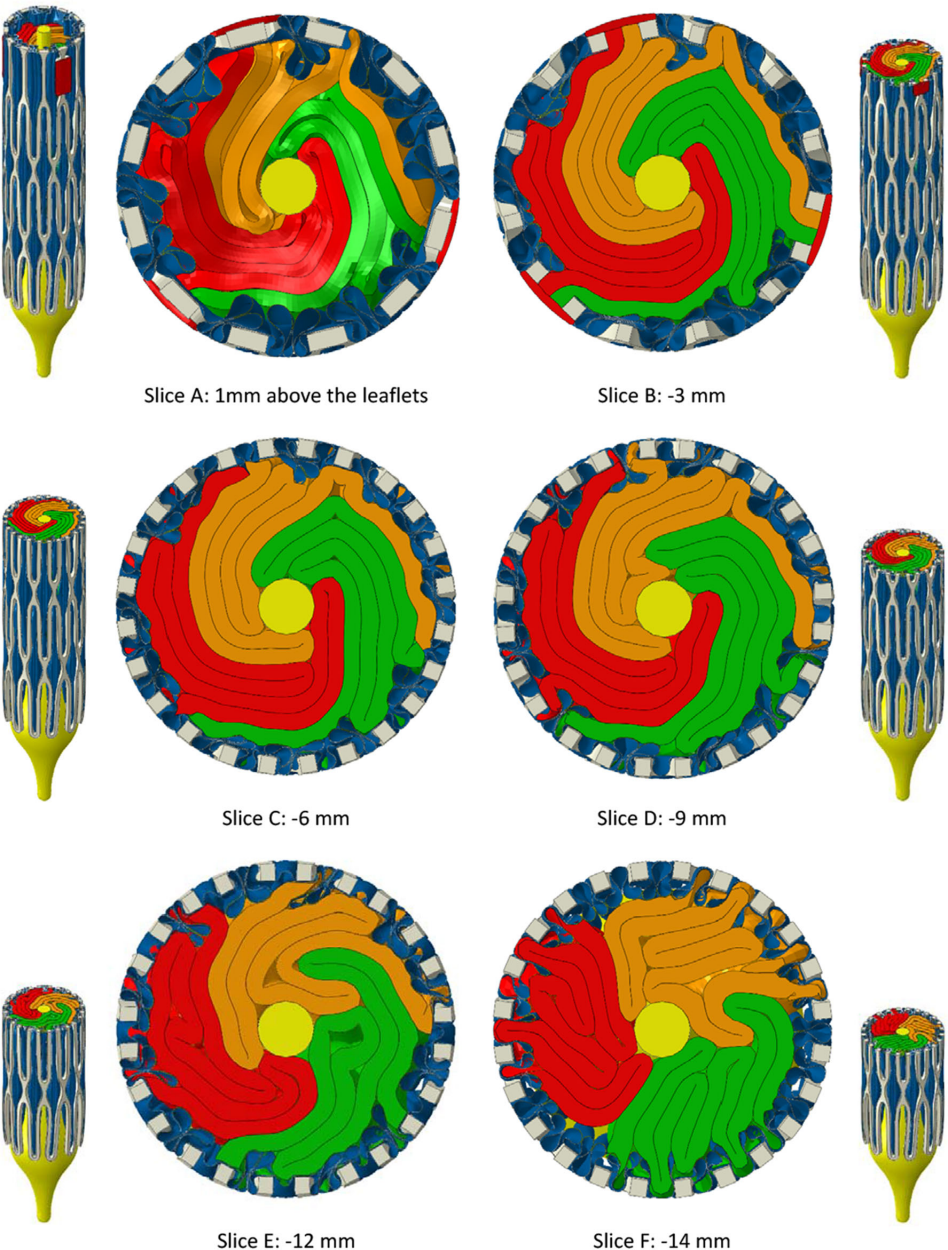
Planar slices through the polymer leaflet model, P300, with 0.30 mm leaflet thickness. The first slice is positioned 1 mm above the top edges of the leaflets. The slices below are spaced at intervals of 3 mm until the last slice—slice F—that is 2 mm below slice E. In this position, the pinching of the leaflets between adjacent frame struts is clear.

The folding patterns of the leaflets are conveyed in Figs. 4 and 5, showing, respectively for B300 and P300, arrays of slices orthogonal to the valve axis in the final crimped state. Isometric views are included showing the positions of the slices. The first slice (A) is 1 mm above the top faces of the leaflets and the other slices are spaced at intervals of 3 mm below slice A apart from slice F which is 2 mm below slice E. This last slice was included since it shows how skirt folds became pinched between adjacent frame struts.

For B300, the leaflets were irregularly folded in what appears to be a clockwise direction with respect to the commissural posts. Relatively tight folds persisted through slices B and C with evidence of them opening by slice D. Whilst there was a certain amount of variability in the skirt folds, they generally had a consistent pattern of interior folds corresponding to the exterior three fold arrangement between frame cells that formed early in the crimping process as seen in Fig. 2.

The same set of slices for P300 in Fig. 5 show that the leaflets folded in an anti-clockwise direction with noticeably stronger rotational symmetry than in B300. Tighter packing of the leaflets is also clear through slices A to D and more folds per leaflet were pinched between frame struts in slice F.

**Figure 6:**
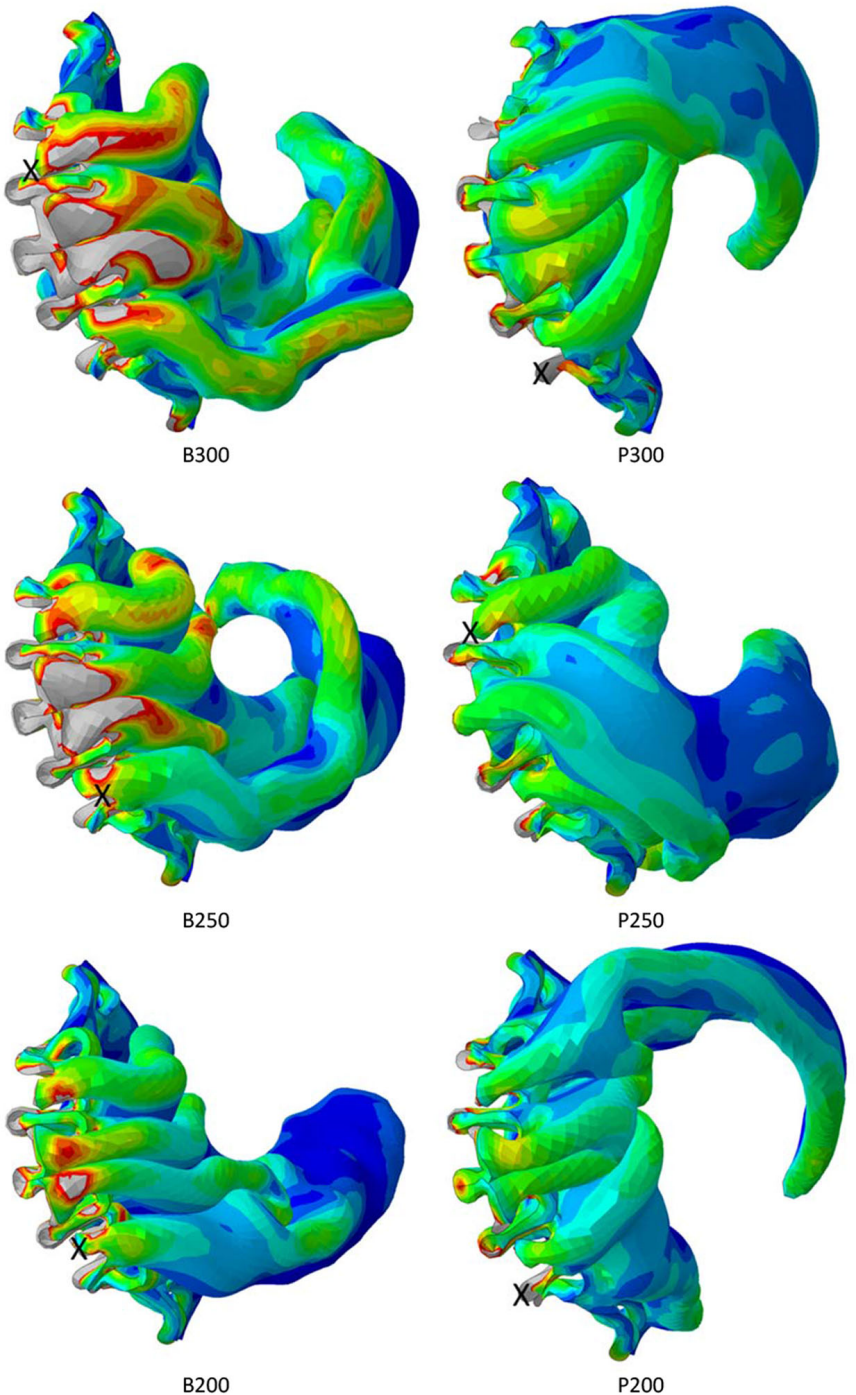
Distributions of von Mises stress in all leaflet models in the final crimped state. A single leaflet is shown viewed from below the valve. A stress threshold of 1 MPa is set in all cases. Higher stresses are clearly shown in the bovine leaflet models and the crosses signify the locations of maximum stress.

Leaflet damage during crimping is most likely to occur in the regions of elevated stress close to the suture lines between the leaflets and the skirt and along the axially aligned spiralling folds from the bases to the tops of the leaflets. Figure 6, shows the von Mises stress distribution in one of the leaflets, as viewed from below the valve, for all six cases. There is some appeal in this projection since it elegantly captures the partially spiralling folding patterns less readily illustrated in the slice projections in previous figures. The spiralling direction was consistent for the bovine leaflets but not so for the polymer cases. A threshold of 1.0 MPa was set to highlight the regions of elevated stress and to qualitatively discern the patterns of stress distribution between the six simulated cases, noting that (i) stresses are visibly smaller and less intense in the polymer leaflets and (ii) the extent of high stresses increases with increasing leaflet thickness in the bovine leaflets. Table 2 lists the peak and average von Mises stresses. In contrast to the bovine models, maximum stress in the polymer leaflets increased with thickness. In all cases, the maximum stress was located close to slice F in one of the suture line folds pinched between adjacent frame struts.

**Table 2:** Maximum and average von Mises stresses for both leaflet models and all thicknesses.

Leaflet model	Maximum von Mises stress, MPa	Average von Mises stress, MPa
B300	9.70	0.285
B250	8.36	0.215
B200	10.99	0.150
P300	4.43	0.227
P250	2.99	0.183
P200	2.59	0.144

**Figure 7:**
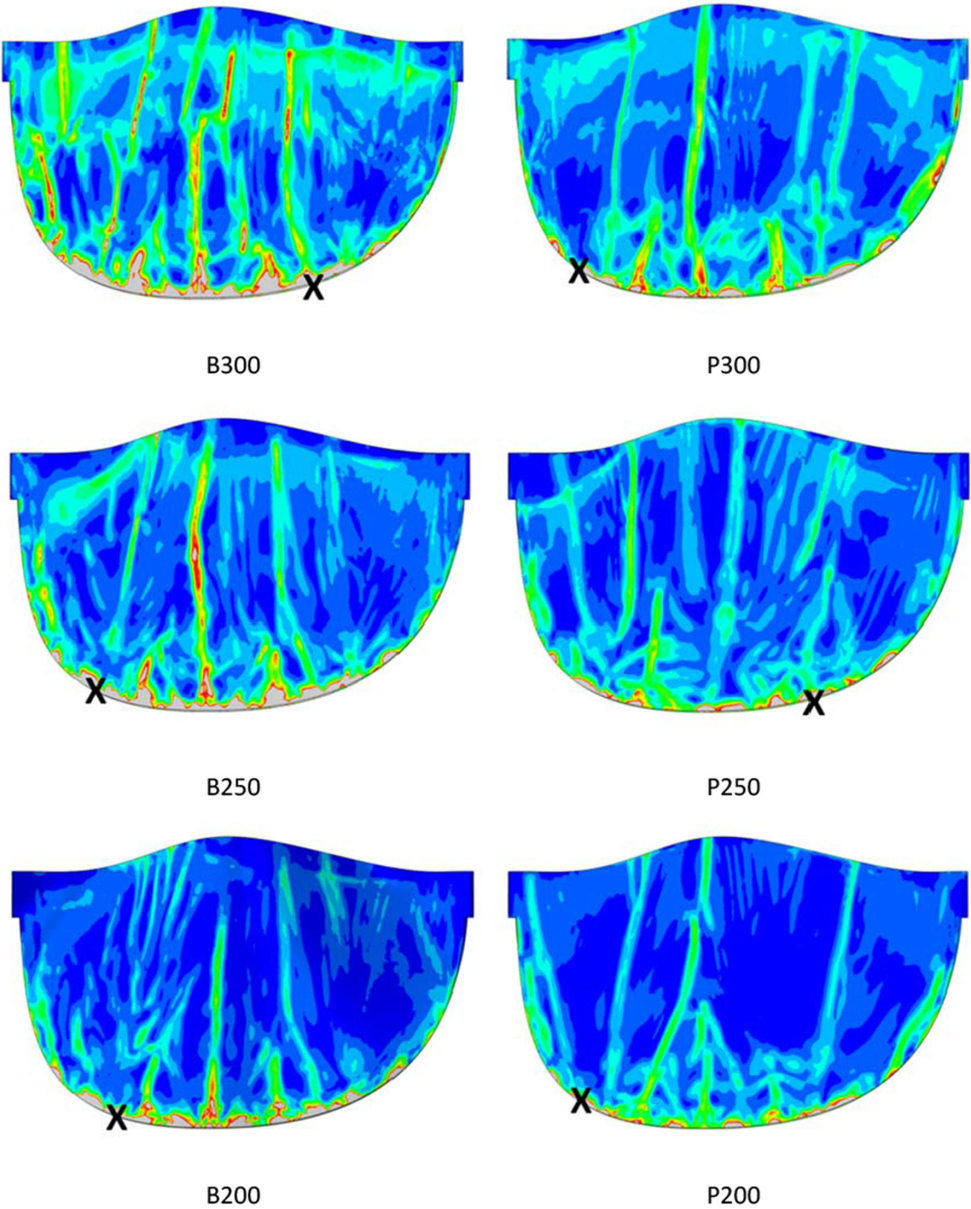
Distributions of von Mises stress in the final crimped state, displayed on the un-deformed leaflet shape. A stress threshold of 1 MPa is set in all cases and the crosses signify the locations of maximum stress.

A clearer illustration of the distribution of leaflet stresses is depicted in Fig. 7 in which the stresses at the end of crimping to 6 mm are displayed on the un-deformed leaflet shape. In addition to the observations already made, these views illustrate two important findings. First, in addition to having higher stresses close to the suture line, the bovine models experienced more intense stresses in the main folds for the B250 and B300 cases. Second, there is a discernible increase in stress intensity and average leaflet stress with increasing leaflet thickness for both leaflet materials resulting from an increasingly tighter packing of the thicker leaflets at the diameter of 6 mm.

**Figure 8:**
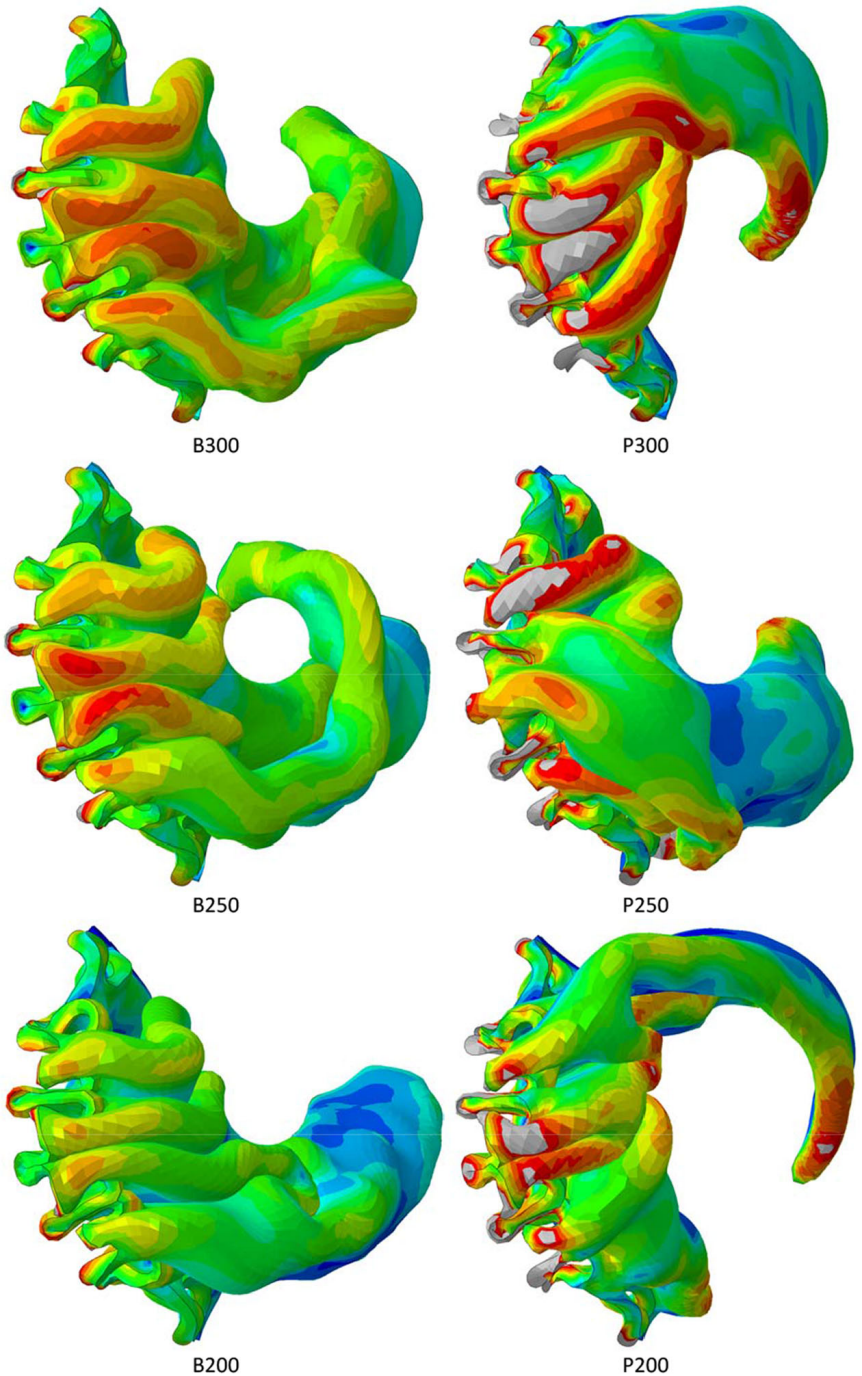
Distributions of logarithmic maximum principal strain in all leaflet models in the final crimped state. A single leaflet is shown viewed from below the valve. A strain threshold of 0.45 is set in all cases.

A more complete understanding of the impact on the leaflets is provided by consideration of leaflet strains. Figure 8 depicts the logarithmic maximum principal strain, similarly for all six cases. Again, an arbitrary threshold was selected—equal to 0.45—to clearly illustrate the range of variation.

## Discussion

### Full Valve Model

To date, computational modelling of transcatheter aortic valve implantation, TAVI, has not included full models comprising a fabric skirt and prosthetic leaflets, in addition to the stent frame, when simulating valve crimping to very small diameters. Indeed, simulation of crimping appears to have been particularly challenging. In this study, these challenges have been overcome to successfully crimp a self-expanding valve to a diameter of 6 mm. The main features of the full model deemed to have made this possible are primarily linked to the treatment of the fabric skirt.

The mesh resolution of 0.05 mm spacing in the skirt was twice the number of elements used by Bailey *et al*.,^6^ over four times the number in Kundail^15^ and many multiples of the number used by Luraghi *et al*.^20^ That said, it is certainly acceptable to use finite strains in coarser meshes but, in the study presented here, it was not possible to successfully simulate the skirt with such meshes when crimping to a diameter of 6 mm.

Successful simulation was sensitive to the effective stiffness of the skirt, determined by the Young’s modulus (50 MPa) and the fabric thickness (0.06 mm) such that no damping was needed. Indeed, no damping was used for any part in the simulation. However, damping may be required for the model when extending it to deployment simulations.

Over two hundred simulations were conducted during the development of the final full model and the successful simulation settings. Most failed simulations resulted from excessive distortion and wave speeds of elements in the skirt. Once working, simulations were conducted to investigate the impact of leaflet thickness on stresses in leaflet models for bovine and polymer materials.

### Peak Leaflet Stresses

In all cases, elevated stresses were produced within the leaflet folds and along the suture lines between the skirt and the leaflets. The largest stresses occurred in the latter locations with maximal values occurring where the leaflets became pinched between adjacent frame struts. For the polymer leaflet model, peak stress increased with leaflet thickness. However, for both leaflet models, average stress increased with leaflet thickness.

This relationship between leaflet thickness and stress shown here runs counter to that predicted during dynamic loading of an opening and closing valve. In a relatively early FEA study of pericardial leaflets, Li and Sun showed how peak stresses close to the commissures of the valve model they used reduced with increasing thickness under pressure loading for mean leaflet thicknesses between 0.20 and 0.35 mm.^19^ Abbasi and Azadani also used FEA to assess the effect of reduced tissue thickness, across a wider range from 0.50 to 0.18 mm, and predicted that, under dynamic loading, the peak stress increased by over 500% during diastole.^1^ Xuan *et al*. summarised the findings of several studies for peak principal stress across a range of leaflets types and sizes.^35^ Values ranged from 0.92 to 2.52 MPa. Further, Gunning *et al*. predicted peak stresses of 2.97 MPa in a self-expanding valve when deployed in an aortic root model.^13^ Again, using FEA, Xuan *et al*. predicted a maximum principal stress of 2.7 MPa in diastole, located at commissural tips where the leaflets were attached to the frame of a 26 mm SAPIEN 3 model.^34^ Slightly lower levels of stress were found in the lower sutured leaflet edges. Across this range of studies, peak leaflet stresses were predicted to be no higher than 3.0 MPa. Thus, the findings in the present study that even higher stresses can occur during crimping to small diameters serves to reinforce the significance of potential leaflet damage during crimping. Leaflet tearing is a recognised problem in bioprosthetic valves and one that could become more significant for valves that are crimped to increasingly smaller diameters. Evidence will be needed to determine whether or not any correlations exist between the locations of fatigue damage and the predicted high stresses close to the suture lines and/or along the folds of leaflets.

Whilst it is difficult to measure leaflet stresses experimentally, several experimental studies have shown how crimping can structurally change/damage leaflets. Kiefer *et al*. used histologic and electron microscopic examinations to show how crimping produced “major changes in the structural morphology of collagen fibres”. They recommended against extensive pre-crimping and cautioned against the tendency towards tighter crimping and its potential to affect valve durability.^16^ Subsequently, Alavi *et al*. observed significant damage in both the surface and deeper layers of pericardial leaflets which worsened with increasingly smaller crimping diameters.^2^ More recently, Xuan *et al*. also cautioned against the impact of elevated leaflet stresses produced in thinner leaflets and how they may lead to earlier leaflet degeneration.^34^ However, it has been shown in the present study that leaflet stresses are not only inversely related to leaflet thickness, as previously demonstrated under conditions of dynamic loading since, in a crimped valve, crimping diameter and the associated volume inside the crimped valve impact both the peak stress and the average stress.

As shown in Fig. 8, the logarithmic maximum principal strains are higher in the polymer leaflets for all three thicknesses. The peak strains are 1.28, 1.00 and 1.07 for thicknesses 3.0, 2.5 and 2.0 mm, respectively. For the bovine leaflets, the peak strains are, respectively, 0.65, 0.63 and 0.56. In all cases, the logarithmic maximum principal strains are higher than the maximum strains reported from the tensile tests conducted by Pfensig *et al*.^28^ and Abassi *et al*.^1^ for polymer and bovine materials, respectively. This suggests that loading on the leaflets with the thicknesses considered here, for the material property models used, could lead to failure in crimping to 6 mm. Under such conditions, a larger minimum crimping diameter would be necessary. Equally, avoidance of failure strains by using thicker leaflets will require a larger minimum crimping diameter due to the limited volume inside the frame when fully crimped.

Consequently, to minimise damage to leaflets, they should not be made too thin, and, whatever thickness is used, potential damage should be minimised by not crimping to such small diameters that lead to tightly folded leaflets and elevated stresses and strains in the folds and along the suture lines between the leaflets and the skirt.

## Limitations

The results presented in this article have been obtained for a self-expanding valve based on a frame design being developed for a new type of transcatheter heart valve. The frame comprises a mix of features from existing commercially available nitinol valves. Currently, no evidence is available to confirm that the results obtained here will be generally applicable to all valve types. However, successful modelling of skirt behaviour during crimping to small diameters is likely to require mesh resolutions close to 0.05 mm.

In common with most, if not all, simulations of TAVI valves, results are dependent on multiple modelling assumptions. In the present work, it will be particularly important to understand the impact on leaflet stress predictions of (i) leaflet shape, material property model specification, mesh type and resolution; (ii) chemical and fixation treatment of the leaflets and (iii) the way constraints and other boundary conditions are defined for the securing of the leaflets to the frame and skirt. In the approach adopted here, the so-called leaflet pads were defined in a way that will be readily repeatable by other researchers. Despite the limitations detailed above, evidence from the research reported in this article suggests that elevated stresses during crimping are likely to always occur in the vicinity of the suture line between the leaflets and the skirt and along the multiple folds that are created in the leaflets.

Also, in common with much previous research, a key limitation of this study concerned the use of radial displacement when, in reality, self-expanding valves are crimped using axial displacement through a tapered funnel. Ongoing research is investigating this approach and the differences that might result with respect to leaflet folding and leaflet stresses.

Finally, the valve design used in this study needs to be optimised, particularly in relation to (i) the shapes of the leaflets and the skirt and (ii) the deployment strategy of the device with due attention for valve anchoring in the annulus and avoidance of coronary obstruction.

## Conclusions

In conclusion, this study has presented a detailed description of the requirements needed to simulate crimping of a self-expanding TAVI device to a small diameter of 6 mm. The full model comprised separate parts for the frame, skirt and leaflets. In particular, a fine mesh was required for the skirt with a spacing of 0.05 mm and small membrane strain elements. Further, the variation of leaflet stresses for different leaflet thicknesses showed average von Mises stress increased with leaflet thickness for both bovine and polymer material property models. Finally, whilst maximum von Mises stress was predicted to be higher in the bovine leaflets, the variation of maximum von Mises stress with thickness was inconsistent between the two material property models.

## Supplementary Information

Below is the link to the electronic supplementary material.

Supplementary file1 (PDF 132 kb)

## References

[1] Abbasi M, Azadani AN (2015). Leaflet stress and strain distributions following incomplete transcatheter aortic valve expansion. J. Biomech..

[2] Alavi SH, Groves EM, Kheradvar A (2014). The effects of transcatheter valve crimping on pericardial leaflets. Ann. Thorac. Surg..

[3] Auricchio F, Conti M, Morganti S, Reali A (2014). Simulation of transcatheter aortic valve implantation: a patient-specific finite element approach. Comput. Methods Biomech. Biomed. Eng..

[4] Auricchio F, Taylor RL (1997). Shape-memory alloys: modelling and numerical simulations of the finite-strain superelastic behavior. Comput. Methods Appl. Mech. Eng..

[5] Bailey, J. Implications for leaflet behaviour in heavily calcified patient-specific aortic roots: simulation of transcatheter aortic valve implantation. PhD Thesis, University of Southampton, 2015.

[6] Bailey J, Curzen N, Bressloff NW (2016). Assessing the impact of including leaflets in the simulation of TAVI deployment into a patient-specific aortic root. Comput. Methods Biomech. Biomed. Eng..

[7] Bailey J, Curzen N, Bressloff NW (2017). The impact of imperfect frame deployment and rotational orientation on stress within the prosthetic leaflets during transcatheter aortic valve implantation. J. Biomech..

[8] Bianchi M, Marom G, Ghosh RP (2019). Patient-specific simulation of transcatheter aortic valve replacement: impact of deployment options on paravalvular leakage. Biomech. Model Mechanobiol..

[9] Bosmans B, Famaey N, Verhoelst E, Bosmans J, Vander Sloten J (2016). A validated methodology for patient specific computational modeling of self-expandable transcatheter aortic valve implantation. J. Biomech..

[10] Capelli C, Bosi GM, Cerri E, Nordmeyer J, Odenwald T, Bonhoeffer P (2012). Patient-specific simulations of transcatheter aortic valve stent implantation. Med. Biol. Eng. Comput..

[11] Carroll JD, Mack MJ, Vemulapalli S, Herrmann HC, Gleason TG, Hanzel G (2020). STS-ACC TVT registry of TAVR. JACC.

[12] Finotello A, Morganti S, Auricchio F (2017). Finite element analysis of TAVI: impact of native aortic root computational modeling strategies on simulation outcomes. Med. Eng. Phys..

[13] Gunning PS, Vaughan TJ, McNamara LM (2014). Simulation of self-expanding transcatheter aortic valve in a realistic aortic root: implications of deployment geometry on leaflet deformation. Ann. Biomed. Eng..

[14] Johnson EL, Wu MCH, Xu F, Wiese NM, Rajanna MR, Herrema AJ (2020). Thinner biological tissues induce leaflet flutter in aortic heart valve replacements. PNAS.

[15] Kandail HS, Trivedi SD, Shaikh AC, Bajwa TK, O’Hair DP, Jahangir A (2018). Impact of annular and supra-annular CoreValve deployment locations on aortic and coronary artery hemodynamics. J. Mech. Behav. Biomed. Mater..

[16] Kiefer P, Gruenwald F, Kempfert J, Aupperle H, Seeburger J, Mohr FW, Walther T (2011). Crimping may affect the durability of transcatheter valves: an experimental analysis. Ann. Thorac. Surg..

[17] Kusner J, Luraghi G, Khodaee F, Rodriguez Matas JF, Migliavacca F, Edelman ER, Nezami FR (2021). Understanding TAVR device expansion as it relates to morphology of the bicuspid aortic valve: a simulation study. PLoS ONE.

[18] Levi, T. S., S. V. Nguyen, N. Benichou, D. Maimon, Z. Yohanan, N. Gurovich, B. Felsen, L. Dadonkina, R. Sharoni and E. Sherman. Prosthetic heart valve. US Patent Application 2020/0107929 A1, 2020.

[19] Li K, Sun W (2010). Simulated thin pericardial bioprosthetic valve leaflet deformation under static pressure-only loading conditions: implications for percutaneous valves. Ann. Biomed. Eng..

[20] Luraghi G, Migliavacca F, Garcıa-Gonzalez A (2019). On the modeling of patient-specific transcatheter aortic valve replacement: a fluid–structure interaction approach. Cardiovasc. Eng. Technol..

[21] Mao W, Wang Q, Kodali S, Sun W (2018). Numerical parametric study of paravalvular leak following a transcatheter aortic valve deployment into a patient-specific aortic root. J. Biomech. Eng..

[22] Mariathas M, Rawlins J, Curzen N (2017). Transcatheter aortic valve implantation: where are we now?. Future Cardiol..

[23] Martin C, Sun W (2017). Transcatheter valve underexpansion limits leaflet durability: implications for valve-in-valve procedures. Ann. Biomed. Eng..

[24] Nappi F, Mazzocchi L, Spadaccio C, Attias D, Timofeva I, Macron L, Iervolino A, Morganti S, Auricchio F (2021). CoreValve vs. Sapien 3 transcatheter aortic valve replacement: a finite element analysis study. Bioengineering.

[25] National Institute for Health and Care Excellence, NICE, Heart valve disease in adults: investigation and management, NICE Guideline scope, https://www.nice.org.uk/guidance/gid-ng10122/documents/draft-scope, 2019. 35143140

[26] Otto CM (2000). Timing of aortic valve surgery. Heart.

[27] Pasta S, Gandolfo C (2021). Computational analysis of self-expanding and balloon-expandable transcatheter heart valves. Biomechanics.

[28] Pfensig S, Arbeiter D, Kohse S, Kaule S, Stiehm M, Grabow N, Schmitz K-P, Siewert S (2019). Development of a constitutive law for numerical simulation of artificial leaflet-structures for transcatheter heart valve prostheses. Curr. Dir. Biomed. Eng..

[29] Rocatello G, De Santis G, De Bock S, De Beule M, Segers P, Mortier P (2019). Optimization of a transcatheter heart valve frame using patient-specific computer simulation. Cardiovasc. Eng. Technol..

[30] Rocatello G, El Faquir N, de Backer O, Swaans MJ, Latib A, Vicentini L, Segers P, De Beule M, de Jaegere P, Mortier P (2019). The impact of size and position of a mechanical expandable transcatheter aortic valve: novel insights through computational modelling and simulation. J. Cardiovasc. Trans. Res..

[31] Rotman OM, Bianchi M, Ghosh RP, Kovarovic B, Bluestein D (2018). Principles of TAVR valve design, modelling and testing. Expert Rev Med Devices.

[32] Sturla F, Ronzoni M, Vitali M, Dimasi A, Vismara R, Preston-Maher G (2016). Impact of different aortic valve calcification patterns on the outcome of transcatheter aortic valve implantation: a finite element study. J. Biomech..

[33] Wu W, Pott D, Mazza B, Sironi T, Dordoni E, Chiastra C (2016). Fluid-structure interaction model of a percutaneous aortic valve: comparison with an in vitro test and feasibility study in a patient-specific case. Ann. Biomed. Eng..

[34] Xuan Y, Dvir D, Wang Z, Mizoguchi T, Ye J, Guccione JM, Ge L, Tseng EE (2019). Stent and leaflet stresses in 26-mm, third-generation, balloon-expandable transcatheter aortic valve. J. Thorac. Cardiovasc. Surg..

[35] Xuan Y, Krishnan K, Ye J, Dvir D, Guccione JM, Ge L (2017). Stent and leaflet stresses in 29-mm second-generation balloon-expandable transcatheter aortic valve. Ann. Thorac. Surg..

