# Supplementary material for: Leaflet Stresses During Full Device Simulation of Crimping to 6 mm in Transcatheter Aortic Valve Implantation, TAVI
Source: Cardiovasc Eng Technol. 2022 Mar 1;13(5):735–50. doi: 10.1007/s13239-022-00614-6 (PMC9616759; doi:10.1007/s13239-022-00614-6)
Supplement: Supplementary file 1 — Supplementary file1 (PDF 132 kb) [file 13239_2022_614_MOESM1_ESM.pdf]

Ongoing research using the same valve model is investigating crimping to 6 mm using axial displacement of a sheath as illustrated in Fig. A.

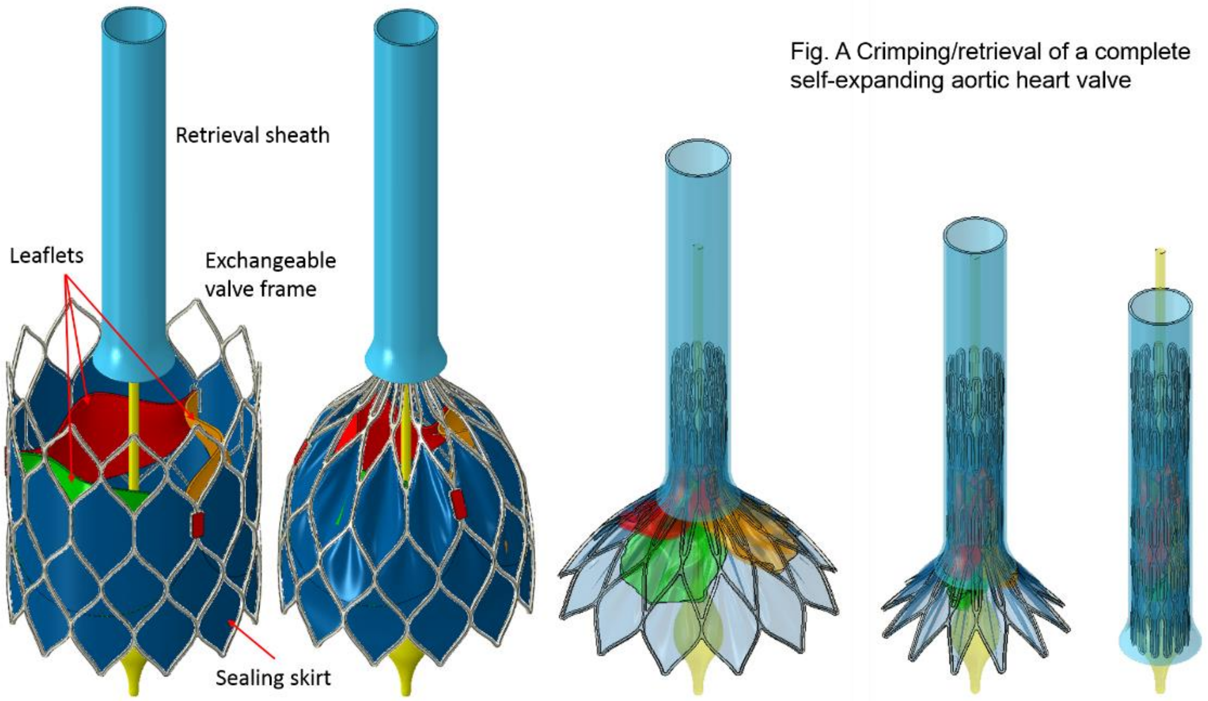

Fig. A Crimping/retrieval of a complete self-expanding aortic heart valve

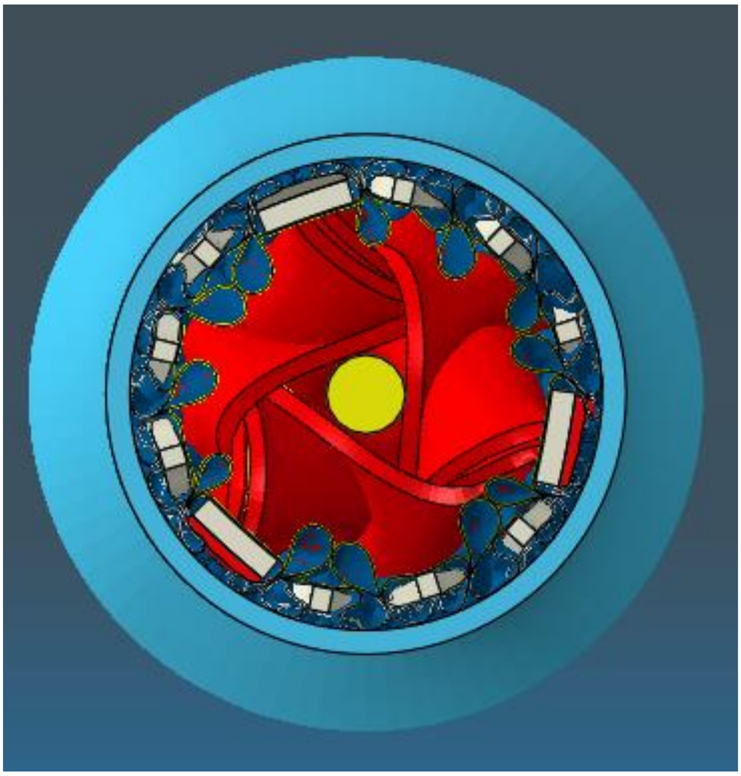

Fig. B View from above illustrating the folding pattern in the leaflets following axial crimping through a sheath.
